# Primary pineal melanoma presenting with leptomeningeal spreading in a 22-year-old woman: a case report

**DOI:** 10.1186/1752-1947-6-165

**Published:** 2012-06-27

**Authors:** Parisa Azimi, Hassan Reza Mohmmadi, Mitra Refiezadeh

**Affiliations:** 1 Department of Neurosurgery, University of Shahid Beheshti Medical Sciences, Imam Hossain Hospital, Shahid Madani Street, Tehran, PA 1617763141, Iran; 2 Department of Pathology, University of Shahid Beheshti Medical Sciences, Imam Hossain Hospital, Shahid Madani Street, Tehran, PA 1617763141, Iran

## Abstract

**Introduction:**

Primary malignant melanoma of the pineal region is exceedingly rare. We report a case of primary pineal malignant melanoma and review the literature.

**Case presentation:**

Our patient was a 22-year-old Iranian woman without any significant past medical history, who was referred to our center with a four-week history of headache and gait disturbance. A magnetic resonance imaging study showed a solid mass in the pineal region causing obstructive hydrocephalus. A brain biopsy was performed and the histological examination indicated melanoma. No other additional melanocytic lesions were found elsewhere. Our patient underwent gross total resection. At the time of discharge she had fully recovered without any neurological deficits. Three weeks after discharge, she was readmitted to hospital with the diagnosis of distal deep vein thrombosis and pulmonary embolism; 12 weeks after the onset of her illness she died of cardiopulmonary arrest.

**Conclusion:**

We have presented here a rare tumor, a primary malignant melanoma of the pineal region. To the best of our knowledge, this is the second-youngest patient with such a tumor reported in the literature.

## Introduction

Primary melanomas of the central nervous system (CNS) are unusual, with an incidence rate of 0.005 cases per 100,000 people [1]. Those in the pineal region are extremely rare and difficult to diagnose [2]. A primary pineal melanoma (PPM) is likely to arise from cells within the leptomeninges surrounding the pineal gland; these cells invade and replace the gland. The outcome for patients with a PPM is poor, particularly in those where it is accompanied by leptomeningeal dissemination [3].

We describe the case of the second-youngest patient reported in the literature with a primary pineal gland melanoma. Some key issues in the diagnosis and treatment, as well as a clinical summary of other patients reported in the literature, are discussed.

## Case presentation

Our patient was a 22-year-old Iranian woman, without any significant past medical history, who presented at another institution with a four-week history of headache, 3 kg weight loss and gait disturbance. She was referred to our center for further evaluation. Our patient's pain was not relieved by pain medications such as aspirin. A week later, her symptoms worsened; she developed lethargy, vomiting, photophobia and incontinence of both sphincters. On neurological examination, our patient was alert and oriented and her speech was fluent and appropriate.

**Figure 1:**
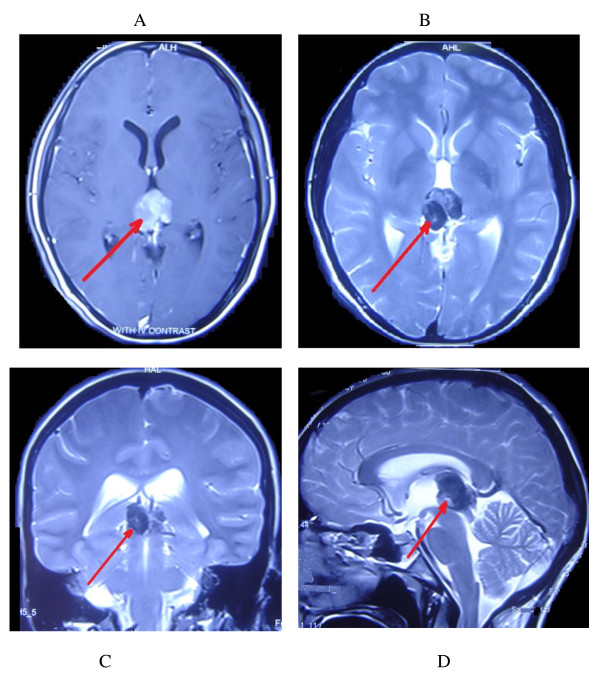
**Preoperative magnetic resonance images showing the mass in the pineal region. (A)** Axial T1-weighted image showing a well demarcated, solid hypersignal mass in the pineal region. **(B, C, D)** Axial, coronal and sagittal T2-weighted images revealing a heterogeneous mass.

Hematologic findings, including a complete blood count, C-reactive protein level, electrolyte levels, erythrocyte sedimentation rate and hepatic enzyme levels, were all normal. Computed tomography showed a hyperdense mass in her pineal region and magnetic resonance imaging (MRI) of her brain revealed a solid mass in her pineal region causing obstructive hydrocephalus. The tumor measured 1.8×1.5×1.7cm (Figure 1A-D).

**Figure 2:**
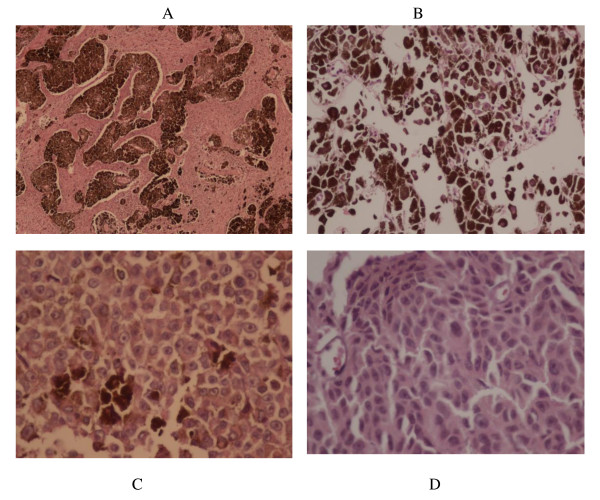
**Histological findings of the pineal mass. (A, B)** Nests of pleomorphic cellular tumor with infiltrative growth pattern containing melanin pigment. **(C, D)** Markedly hypercellular malignant melanoma with hyperchromatic pleomorphic nuclei and prominent nucleoli.

A stereotactic biopsy of her pineal region was performed using a right frontal trajectory. Histological sections revealed a pigmented tumor composed of large polygonal cells with epithelioid morphology, pleomorphic nuclei, prominent eosinophilic nuclei, abundant intracytoplasmic melanin pigment deposition and occasional mitotic figures with necrotic areas (Figure 2A-D). In an immunohistochemical study, the tumor cells displayed strong immunoreactivity for human melanoma black-45 and melan-A.

Ophthalmologic and dermatologic examinations as well as thoracic and abdominal scans uncovered no evidence of extracranial disease. The diagnosis of our patient was based on appropriate imaging studies, assessment by cytopathology and ruling out differential diagnoses.

Five days after admission, our patient underwent a craniotomy. A gross total resection of the tumor through a supracerebellar infratentorial approach and external ventricular drainage was performed. A black pigmented solitary tumor was seen with leptomeningeal dissemination.

Her postoperative length of stay in our intensive care unit was two weeks due to her developing pneumonia. Several repeat MRI scans of our patient’s brain showed no change in the pineal mass or further tumor dissemination. Our patient was discharged from the hospital three weeks after surgery. At the time of discharge she had fully recovered without any neurological deficits. Three weeks after discharge, she was readmitted with the diagnosis of distal deep vein thrombosis in her left leg. During treatment with anticoagulant therapy, our patient was diagnosed with a pulmonary embolism and was transferred to our intensive care unit. She died from a cardiopulmonary arrest four days later, having survived only 12 weeks from the start of her initial symptoms.

The features of the reported examples of PPMs in the literature are summarized in Table 1, along with the findings from our patient.

**Table 1:** Summary of 17 reported cases of primary pineal melanoma

**Author**	**Year**	**Sex**	**Age (years)**	**Time to diagnosis (weeks)**	**Symptoms**	**Imaging**		**Melanic pigment**	**Treatment**	**Survival (weeks)**
						**CT**	**MRI**			
Ogle [4]	1899	F	32	3	Headache, paralysis, aphasia	-	-	NR	None	13
Stoerk	1904	M	31	8	PS, headache, diplopia	-	-	NR	None	12
Foot	1931	M	49	2	Headache	-	-	present	None	4
Gibson	1952	F	68	8	Headache, vomiting, coma	-	-	present	None	8
Enriquez	1973	M	43	32	Left-sided weakness, changes in character	-	-	present	None	37
Arland	1977	M	56	40	PS, gait disturbance, memory impairment	-	-	present	Radiation	56
Carlson	1987	F	77	1	Gait disturbance, poor memory	-	-	NR	VPS, craniotomy, biopsy	5
Weindling	1988	M	59	2	Headache, vomiting, papilledema	+	+	present	Biopsy	NR
Rubino	1992	M	60	4	Gait disturbance, lethargy, diplopia, retraction nystagmus	+	+	present	Radical resection	>52
Yamane	1994	F	60	2	Headache, PS	+	+	present	Resection, chemotherapy	>280
Mitchell [6]	1998	M	49	12	Vomiting, weight loss, drowsiness	+	+	present	Biopsy	NR
Suzuki	2001	F	50	16	Poor memory	+	+	present	Partial resection, radiation	88
Bookland	2006	F	20	3	Headache, cervical pain	+	+	present	Biopsy, VPS, radiation and chemotherapy	>37
Barron	2007	F	73	NR	Headache, gait unsteadiness, double vision, memory change	+	+	present	Radiation	69
Arantes [3]	2010	F	54	16	Gait disturbance, memory changes, lethargy, sphincters incontinence	+	+	present	Biopsy, VPS, resection, radiation and chemotherapy	>80
Cedeño Díaz [5]	2011	M	70	24	Headache, gait disturbance, lethargy, diplopia	+	+	present	Partial resection, radiation	40
Present case	2011	F	22	5	Headache, gait disturbance	+	+	present	Biopsy , radical resection, external ventricular drain	7

CT: computed tomography, F: female; M: male; NR: not reported; PS: Parinaud’s syndrome; VPS: ventriculoperitoneal shunts.

## Discussion

To the best of our knowledge, only 16 cases of primary melanoma arising from the pineal gland have been reported previously in the English literature [3-6]. PPMs constitute nearly 3.6% of primary melanomas in the CNS [7]. Almost all cases are associated with meningeal dissemination [7], similar to our patient. The mean age of patients has been reported to be 51.3 years (ranging from 20 to 77 years), with an equal sex distribution. On average, patients have presented to hospital 10 weeks after the onset of complaints (ranging from one to 40 weeks). Most patients present with symptoms of increased intracranial pressure or superior tectal compression.

Overall, the mean survival has been reported to be 55 weeks from the onset of symptoms, with one case of long-term survival extending over five years. Those patients who get no treatment have the shortest survival time.

CNS melanomas are quite rare. These tumors may occur *de novo* or in association with neurocutaneous melanosis. Primary CNS melanomas have a pathology indistinguishable from that of melanomas arising from the skin, eye or other mucosal sites [8]. A meticulous search for a cutaneous, mucosal or ocular primary melanoma should prove unrevealing before a malignant melanoma can be accepted as indigenous to the CNS [8].

Although MRI is the gold standard for imaging a patient with a suspected PPM [3,5], this tumor cannot be reliably distinguished from metastatic melanoma by neuroimaging alone. The tumor is frequently hypointense in T1-weighted MRI and hyperintense in T2-weighted MRI; however, these typical features can be lacking in some cases [9]. PPM is generally a diagnosis of exclusion, after ruling out extracranial disease in the skin, mucosa or eye in addition to histopathological confirmation from the brain specimen. The correct diagnosis of our patient was based on the MRI findings described by Woodruff *et al*. [10], assessment by cytopathology and, finally, by ruling out an extracranial source.

A safe initial diagnosis without delay in PPM is difficult because of its location, nature and correlation with other structures of the area, which was the case in our patient. The successful treatment of PPM is controversial; however, combined therapy including surgery, radiation therapy and chemotherapy seems to improve the survival rate [3].

Surgical removal has been attempted in eight out of 16 cases reported in the literature. In six cases (38%) total or partial resection was performed; in two cases (12%) no surgical attempt was made; and in five cases (31%) there was no mention of the surgical procedure. Our patient underwent surgery alone and, since we removed the whole tumor, did not receive any radiotherapy regimen.

The outcome in PPM with leptomeningeal dissemination is very poor [3]. Our patient died 49 days after surgery. This short survival might be due to several factors. We suspect our patient died due to physical inactivity after her surgery, causing deep vein thrombosis and a pulmonary embolism. To the authors’ best knowledge, no evidence-based guidelines exist for the treatment and management of these melanomas. However, in reported cases, combination therapy has shown longer survival than other methods.

## Conclusion

We have presented here a rare tumor, a primary malignant melanoma in the pineal region. To the authors’ best knowledge, this is the second-youngest patient reported in the literature.

## Consent

Written informed consent was obtained from the patient for publication of this case report and any accompanying images. A copy of the written consent is available for review by the Editor-in-Chief of this journal.

## Competing interests

The authors declare that they have no competing interests.

## Authors’ contributions

All authors were involved in designing of the study, data collection and analysis, interpretation of results and manuscript preparation. PA prepared the first draft of the paper. HRM and PA provided the final manuscript. All authors read and approved the final manuscript.
